# Bridging awareness and behavior: decoding implicit metacognitive behaviors in AI-assisted programming via fine-grained log analysis

**DOI:** 10.3389/fpsyg.2026.1790196

**Published:** 2026-04-10

**Authors:** Xiaojing Hou, Zhichun Liu, Guoqiang Wang, Nianfeng Shi

**Affiliations:** School of Computer Science, Luoyang Institute of Science and Technology, Luoyang, China

**Keywords:** generative AI in education, human–AI interaction, metacognitive scaffolding, process data analysis, programming education, self-regulated learning

## Abstract

While generative artificial intelligence offers transformative potential for programming education, its impact on students' internal cognitive and behavioral patterns remains underexplored. This study aims to address this “black box” issue by investigating how AI-driven interventions influence metacognitive regulation and self-regulated learning. A randomized controlled trial was conducted with 122 Computer Science undergraduates (mean age = 19.6 years; 28.2% female) from a university in China. Participants were assigned to an AI-assisted intervention group (*n* = 62) or a control group (*n* = 60) within a Python programming course. Using a customized Jupyter environment, an integrated autonomous AI agent monitored real-time behavioral logs and triggered non-directive, process-oriented prompts based on specific algorithmic thresholds. Data collection integrated fine-grained log analysis with standardized assessments to quantify implicit planning, monitoring, and regulation processes. The AI intervention significantly optimized learning behaviors, facilitating a shift from impulsive “trial-and-error” approaches to deliberate planning and superior debugging precision. These behavioral improvements were accompanied by significant gains in both academic performance and subjective metacognitive awareness compared to the control group. The findings confirm that when designed as a process-oriented scaffold, AI functions as a catalyst for self-regulated learning rather than a passive crutch. This study highlights the role of AI as a psychological scaffold that supports metacognitive regulation, providing an evidence-based blueprint for the design of effective learning environments in educational psychology.

## 1 Introduction

With the growing integration of artificial intelligence (AI) into education, utilizing AI to enhance students' self-directed learning has become a core topic in educational reform. Learning to program is a complex cognitive task that highly relies on problem-solving and self-regulation; consequently, outcomes largely depend on students' metacognitive abilities. Research indicates that while metacognitive awareness correlates with success, novices often begin coding prematurely without sufficient planning, leading to inefficient learning processes (Prasad and Sane, 2024; Sun et al., 2025).

In instructional practice, a paradox persists: students often demonstrate high metacognitive awareness in questionnaires but fail to transfer these skills into actual tasks. We hypothesize that this discrepancy stems from the disconnect between metacognitive awareness (static knowledge of cognition) and metacognitive behavior (dynamic enactment of regulation). In programming, merely possessing strategic knowledge is insufficient. Students must translate awareness into active behaviors, such as pre-coding planning and real-time monitoring, which constitute key phases in the self-regulated learning (SRL) cycle. High-performing students typically bridge this gap by dedicating time to planning and debugging, whereas struggling students often resort to blind “trial-and-error.”

Recent advancements in generative AI (GenAI) offer new opportunities to bridge this disconnect between metacognitive awareness and actual self-regulatory behavior during programming. Early-stage learners often possess the declarative knowledge of how they should regulate their learning, yet frequently fail to enact these strategies when facing complex cognitive loads, such as persistent runtime errors (Tuononen et al., 2022; Musullulu et al., 2025). By functioning as a dynamic, context-aware metacognitive scaffold, GenAI can effectively address this “awareness-behavior” gap. Unlike static pedagogical tools, large language models (LLMs) can analyze real-time behavioral traces to deliver personalized prompts when maladaptive behaviors occur. This scaffolding translates inert metacognitive awareness into actionable regulatory steps, guiding students to pause, reflect, and strategize before modifying code. By offering process-oriented feedback rather than direct solutions, AI agents effectively scaffolding the entire learning cycle. Prior research indicates that interactive feedback driven by LLMs can enhance learners' motivation and metacognitive strategies, leading to marked improvements in self-regulated learning and academic performance (Ewell et al., 2023; Yan et al., 2025).

However, existing empirical studies on AI-supported learning environments predominantly evaluate intervention effectiveness through outcome-based indicators such as academic performance, while the underlying learning processes remain comparatively underexplored (Zhai et al., 2021). Few studies have directly verified whether such interventions induce behavioral changes during the learning process (Tsakeni et al., 2025). To address this gap, this study constructed an AI-assisted metacognitive scaffolding model and conducted a randomized controlled trial. We aim to examine two specific hypotheses: (1) AI-assisted scaffolding will significantly improve students' process-oriented metacognitive behaviors compared to unassisted peers; and (2) these behavioral improvements will translate into superior academic outcomes. By verifying these mechanisms, we hope to validate process-oriented evaluation as a means to cultivate “learning how to learn” in the AI era.

## 2 Literature review

### 2.1 Metacognitive processes and behavioral performance in programming

Metacognition, first proposed by John H. Flavell, refers to “cognition about cognitive phenomena”—the ability to recognize and regulate one's own cognitive processes (Flavell, 1979; p. 906). Given that it involves both descriptive knowledge and control strategies regarding cognitive processes, researchers typically conceptualize metacognition through two perspectives: metacognitive knowledge and metacognitive skills (Veenman, 2015). Metacognitive knowledge pertains to an individual's knowledge about cognitive processes, whereas metacognitive skills (sometimes termed metacognitive regulation) involve the ability to actively monitor and regulate thought processes during actual cognitive activities (Stephanou and Mpiontini, 2017). Beyond this foundational view, contemporary SRL frameworks further embed metacognitive processes within a broader, cyclical learning system. In the SRL model proposed by Zimmerman, metacognitive planning, monitoring, and self-evaluation function as core regulatory mechanisms across the forethought, performance, and self-reflection phases of learning (Zimmerman, 2000). In this study, self-reported metacognitive knowledge and skills are collectively referred to as metacognitive awareness, distinguishing them from metacognitive behavior.

Metacognitive behavior refers to the practical enactment of metacognitive awareness, conditioned by multiple factors such as memory capacity, motivation, and habit (Ma et al., 2025). Central to this are three core strategies: planning, monitoring, and regulation (Flavell, 1979; Kouhpayehzadeh Esfahani et al., 2022). In programming tasks, planning involves pre-coding preparation, such as analyzing requirements and outline algorithms. Monitoring implies the ongoing assessment of one's understanding, including logic checks and output verification. Finally, regulation concerns the adaptive adjustment of strategies, such as debugging or consulting resources, to resolve difficulties encountered during the task.

Building upon this theoretical foundation, it is critical to examine the predictive power of these dimensions regarding learning outcomes. Several empirical studies published in recent years have confirmed the positive correlation between the two. For instance, Gura et al. reported a robust and statistically significant correlation (*r* = 0.709) between metacognitive awareness and behavioral regulation among professionals, suggesting that individuals with a deeper understanding of cognitive processes tend to be more inclined to use moderating strategies in practice (Gura et al., 2020). Similarly, Bubnic et al. (2024) employed structural equation modeling (SEM) to analyze data from 194 students enrolled in an object-oriented programming course. Their core findings are particularly relevant to our study: although metacognitive awareness is positively correlated with academic performance, awareness alone is insufficient to drive significant improvement. Crucially, it is the specific metacognitive behaviors exhibited by students that account for 27% of the variance in programming outcomes (Bubnic et al., 2024).

These findings provide a critical direction for our research in programming education. Traditional pedagogy often focuses on raising “awareness,” where instructors emphasize principles such as “strategic planning prior to coding” or “adherence to coding standards.” However, if these verbal instructions fail to translate into consistent behavioral habits, their educational efficacy will be greatly compromised. We need mechanisms that not only encourage but ensure students perform specific metacognitive tasks. This creates a unique opportunity for GenAI to serve as a behavioral scaffold, transforming passive knowledge into active regulation.

### 2.2 Scaffolding and AI-assisted programming instruction

Scaffolding is defined as an instructional support mechanism where teachers or more experienced individuals provide support and guidance to learners who are unable to accomplish tasks independently, thereby enabling them to bridge the gap to autonomous mastery (Wood et al., 1976). Scaffolds are typically categorized by functions. Cognitive scaffolds help in knowledge acquisition and problems decomposition, whereas metacognitive scaffolds operate at the “thinking of thinking” level. The latter guides students to reflect on their learning approach, fostering active regulation of their cognitive processes (An and Li, 2014).

With the popularity of LLMs like ChatGPT, the feedback mechanism in programming education has undergone a revolutionary change (Tikva and Tambouris, 2023). Traditional automated assessment tools typically provide outcome-focused feedback limited to execution results or on syntax errors, often failing to address the underlying cognitive processes of learners (Beale, 2025). In contrast, LLMs-based systems have the ability to comprehend and generate natural language, enabling the provision of process-oriented, scaffolded guidance (Liu et al., 2026).

Recent pedagogical innovations have increasingly leveraged LLMs to act as metacognitive agents. Approaches vary from simulating human tutor interactions to structuring problem decomposition via “learner-LLM co-decomposition” (Ma et al., 2025; Sun et al., 2025). Beyond simple interaction, data-driven frameworks like CAELF and Co-Learning further demonstrate that LLM-generated feedback can serve as a fundamental scaffold for reflection, effectively activating students' time management and attention regulation (Hong et al., 2024; Yu et al., 2025).

Collectively, these studies highlight a critical pivot: there is a compelling need for GenAI to transit from passive “writing aid” to an active “external metacognitive agent” to mitigate the risk of becoming a mere crutch (Menon, 2025; Tsakeni et al., 2025). This shift is particularly crucial for addressing the cognitive overload that often causes beginning programmers to bypass essential monitoring processes. As explained by cognitive load theory (CLT), the heavy cognitive demands of programming can easily overwhelm a novice's limited working memory, depleting the cognitive resources necessary for higher-order metacognitive regulation (Sweller, 2011). However, while theoretical models suggest that such AI scaffolding can bridge the gap between awareness and behavior, empirical evidence verifying these micro-behavioral changes remains scarce in the GenAI context. To address this gap, the present study employs a randomized controlled trial to empirically validate these dynamics and clarify the mechanism of AI-assisted scaffolding.

## 3 Theoretical framework: modeling metacognitive behaviors in programming

Our model design builds upon the “Generated and Adjusted Feedback Metacognitive Scaffolding (GAF-MetaScaffolding)” framework established in our prior work (Hou et al., 2026). While the original comprehensive framework explores collaborative scaffold construction, this study strictly extracts and operationalizes its “Behavior Feedback Module” to specifically address students' cognitive processes during actual programming practice. This adaptation directly targets the core phases of SRL—planning, monitoring, and regulation—aiming to mitigate cognitive load and counteract metacognitive inertia through timely interventions.

**Figure 1:**
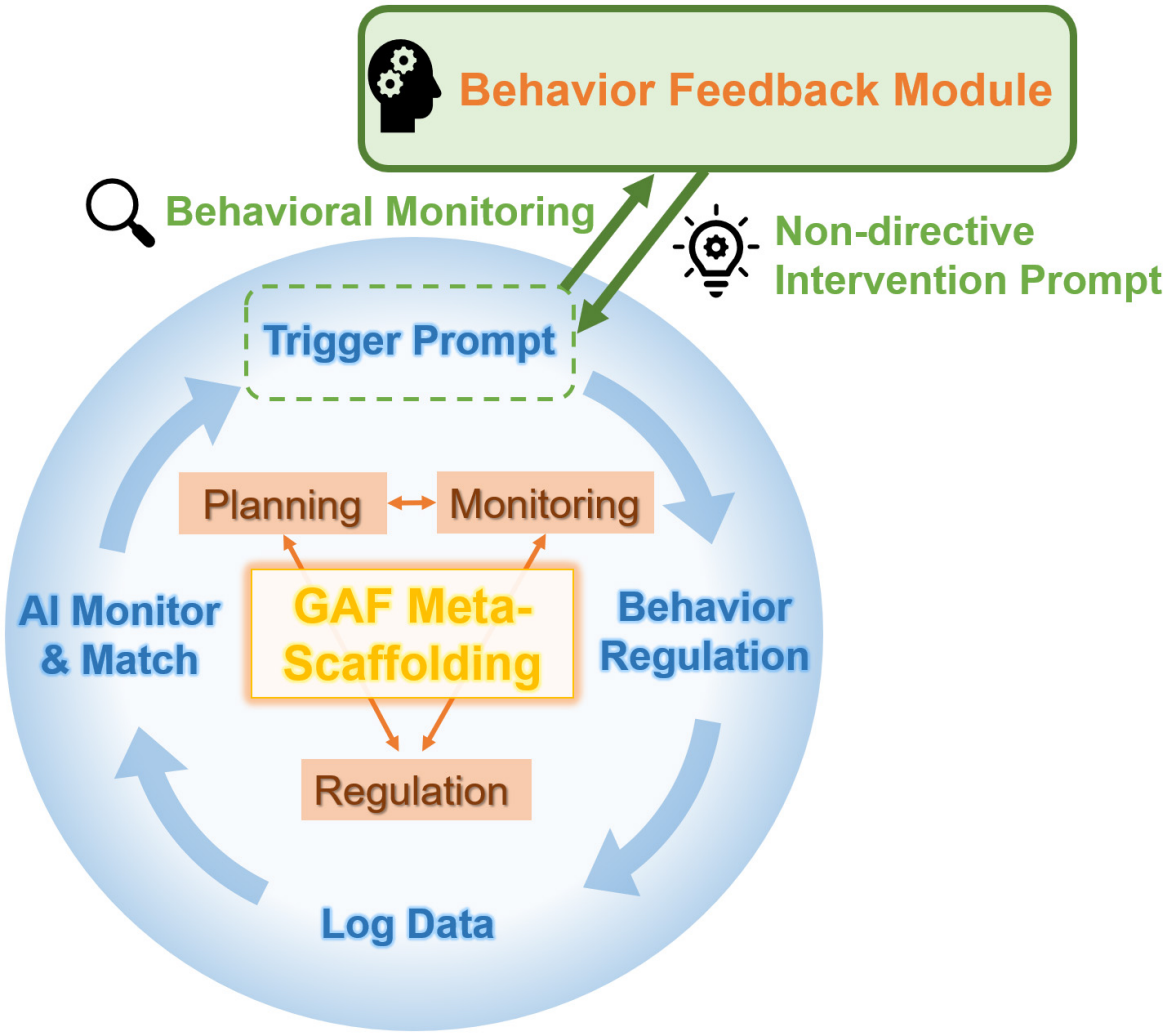
Conceptual architecture of the autonomous behavior feedback loop. Adapted from the GAF-MetaScaffolding framework, this continuous cycle integrates real-time log data monitoring with targeted, non-directive intervention prompts to dynamically scaffold students' core metacognitive processes (planning, monitoring, and regulation) during programming.

As illustrated in the enhanced conceptual model (Figure 1), the system operates on a continuous, four-stage autonomous loop. First, as students engage in programming tasks, their coding activities continuously generate fine-grained Log Data. Second, the system employs an AI Monitor & Match mechanism to analyze these behavioral traces in real-time, comparing them against predefined algorithmic thresholds. When a maladaptive pattern is detected, the Behavior Feedback Module actively intervenes. It executes a Trigger Prompt, which is deliberately designed as a non-directive intervention prompt. For instance, rather than providing direct code solutions, the system unobtrusively prompts: “try running your code to check if the current output matches your expectations?” or “consider reviewing the latest error prompts to explore potential causes” (Çakiroglu and Er, 2023). Ultimately, these lightweight prompts function as external metacognitive cues that facilitate Behavior Regulation. They guide students to pause, reflect, and consciously adjust their internal cognitive strategies before continuing the task. To ensure immediacy and consistency, the prompts in this study are automatically triggered by predefined algorithmic thresholds. In this way, the system provides timely support that helps translate students' metacognitive awareness into actual regulatory behaviors.

**Figure 2:**
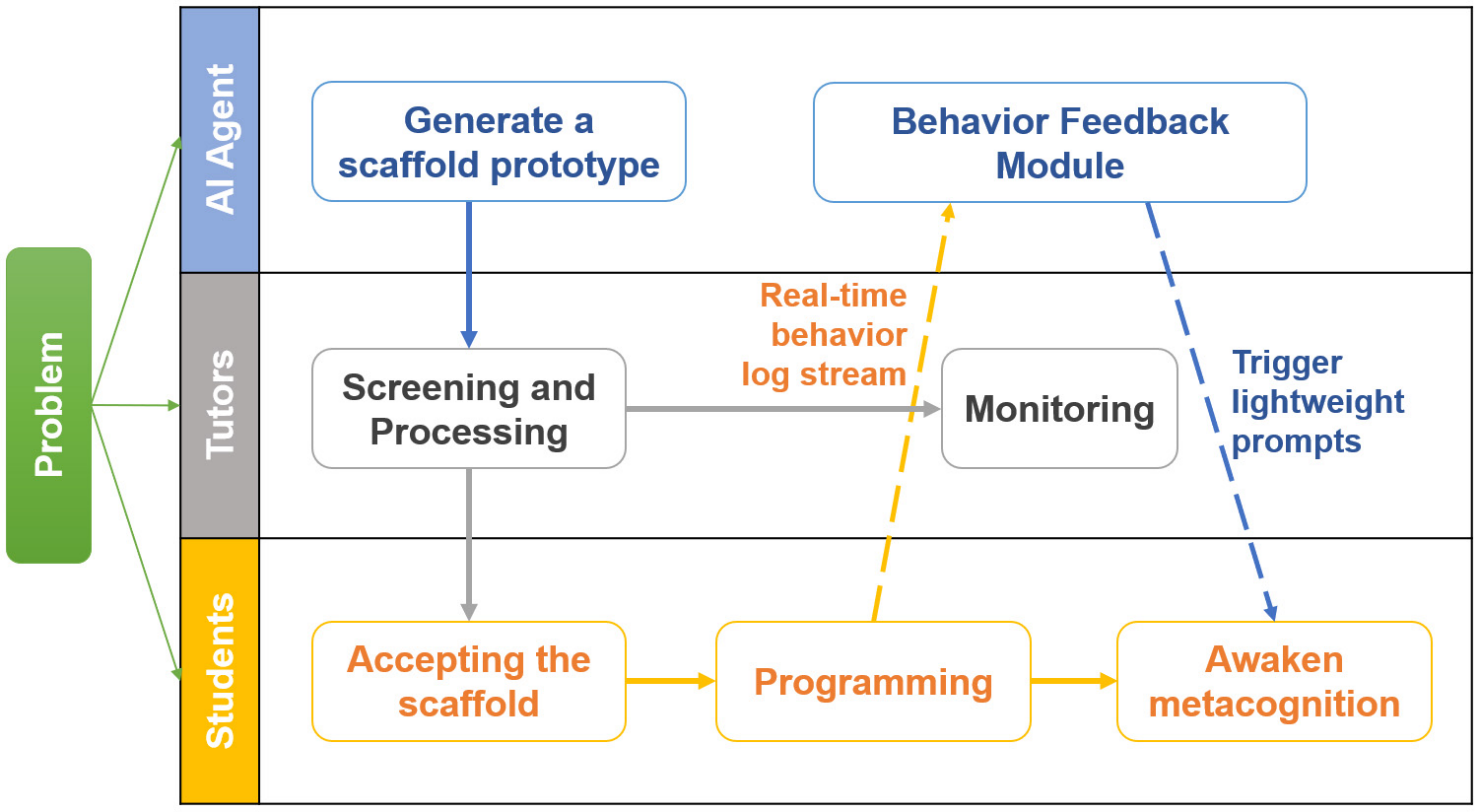
Interaction Swimlane diagram illustrating the AI-assisted scaffolding process. The diagram delineates the distinct roles of the AI agent, tutors, and students. Solid lines indicate the instructional workflow, while dashed lines represent the data flow of behavioral logs and triggered prompts. The process highlights how the system transforms passive log data into active, lightweight metacognitive interventions (e.g., pop-ups) without disrupting student autonomy.

Distinct from code-generation tools, this module provides process-oriented guidance. It also promotes self-reflection by displaying behavioral data, such as “time since last run” or “error frequency,” directly on the dashboard. Figure 2 maps this interaction flow, clearly delineating the distinct roles of the AI, tutors, and students. The solid lines in the diagram represent the specific experimental workflow, while the dashed lines indicate the flow of data. By prioritizing behavioral guidance over direct solution provision, this approach preserves opportunities for autonomous problem-solving while ensuring that students' metacognitive processes are actively supported.

In summary, our AI-assisted scaffolding framework aims to cultivate sustainable learning habits and problem-solving strategies through timely metacognitive support. This design addresses the limitations of existing systems regarding micro-behavioral intervention. By prioritizing the student's behavioral process over mere academic performance, this study explores the potential of AI to fundamentally promote metacognitive development.

## 4 Materials and methods

### 4.1 Participants and procedure

This study employed a randomized controlled trial design, recruiting participants through convenience sampling from undergraduate students (total of 126) enrolled in a Python programming course, majoring in Computer Science or related fields. Participant recruitment was conducted between January 2025 and February 2025, prior to the commencement of the academic semester. To control for confounding variables, specific eligibility criteria were applied: all participants had to be full-time students, first-time enrollees in the course, and have no prior formal programming experience. Participants were randomly assigned to either the AI-assisted or control group using a random sequence generated by the =RAND() function in Microsoft Excel. Before the intervention, all students completed a uniform 6-week introductory module covering foundational Python syntax to ensure an equal baseline of prior knowledge, including variables, loops, conditional statements, and basic data structures. Following this instruction, they completed pre-tests, including a questionnaire for baseline metacognition and a coding task to measure initial programming ability. To prevent practice effects while ensuring assessment reliability, the study employed isomorphic programming tasks across the pre-test, intervention, and post-test phases.

Each task required students to process a structured text dataset (approximately 200–300 records) and generate summary outputs. The tasks involved similar algorithmic structures and programming constructs, including file input/output operations, string parsing, loop-based data processing, dictionary or list data structures, conditional filtering, and result aggregation. Typical solutions required approximately 50–80 lines of code. Although the tasks differed in contextual scenarios to minimize memorization effects, they imposed comparable cognitive demands and covered the same core Python concepts. For example, the pre-test task required analyzing product sales records, while the post-test task involved reading employee attendance logs and identifying anomalous attendance patterns. Programming solutions were evaluated using a standardized rubric.

During the intervention phase, clear distinctions were established between the two experimental conditions. For the AI-assisted group, the intervention was rooted in Zimmerman's self-regulated learning theory and the GAF-MetaScaffolding framework (Zimmerman, 2000; Hou et al., 2026). Students completed tasks within a customized Jupyter Notebook environment integrated with an AI agent via a customized extension and API hooks. In terms of implementation, this AI did not function as a code-generation tool; rather, it provided proactive, real-time metacognitive scaffolding. To ensure the transparency and replicability of the intervention, abstract scaffolding strategies were operationalized into specific rule-based triggers (detailed in Table 1). The system continuously monitored behavioral logs and triggered non-directive pop-up prompts (e.g., suggesting a review of requirements or analyzing traceback errors) only when specific maladaptive behaviors were detected. These triggers are firmly grounded in empirical research on novice programming and SRL theory (Jadud, 2006; Loksa et al., 2016):

- The “Impulsive Start” trigger addresses the common novice tendency to bypass critical problem representation and planning phases, a habit known to severely hamper debugging efficiency.
- The “Lack of Verification” condition targets the maladaptive practice of writing extensive code without intermittent testing, which compounds logic errors.
- For the regulation phase, the “Ineffective Trial” trigger algorithmically captures rapid and blind edits indicating a breakdown in metacognitive monitoring. Conversely, the “Stagnation” trigger addresses students who stop when facing cognitive impasses.

**Table 1:** Examples of behavioral triggers and AI-generated metacognitive prompts.

Metacognitive phase	Behavioral trigger condition	Prompt example
Planning	Impulsive start: first keystroke occurs <60 s after opening the task.	“You started coding very quickly. Have you broken down the problem requirements? Try writing a few lines of pseudocode comments first.”
Monitoring	Lack of verification: no code execution event for >15 min while active editing continues.	“It has been a while since your last run. Would you like to execute the current code block to verify if the intermediate output matches your expectations?”
Regulation	Ineffective trial: three consecutive “error” results with edit distance <3 lines (blind retries).	“Repeated errors detected. Instead of guessing, consider reading the last traceback message carefully to identify the logic flaw.”
	Stagnation: idle time >10 min without any edit or execution.	“It seems you are stuck. Try reviewing the task description again or checking the documentation for the specific function you are using.”

By translating these well-documented behavioral patterns into precise algorithmic thresholds, the AI interventions are both theoretically justified and pedagogically timely.

For the control group, students relied solely on a “standard tool,” which is strictly defined in this study as a traditional pedagogical baseline: the native Jupyter Notebook IDE. Specifically, the control group environment provided standard syntax highlighting, code execution, and native Python runtime error tracebacks. Importantly, to strictly isolate the effect of metacognitive scaffolding and avoid confounding variables, the control group had absolutely no access to proactive AI pop-ups or conversational generative AI assistants during the tasks. Regardless of the assigned condition, all participants worked within the Jupyter ecosystem, with their fine-grained behavioral log data recorded unobtrusively in the background to ensure high ecological validity.

Following the intervention, all participants completed a post-test comprising three components: (1) a programming problem equivalent in complexity to the intervention tasks but in a different scenario; (2) a post-test metacognitive questionnaire; and (3) the submission of programming process logs and source code. Over the 21-day experiment, we collected comprehensive data including survey responses, code artifacts, and behavioral logs. The study was conducted in strict accordance with ethical standards regarding voluntary participation and data privacy.

### 4.2 Measures

#### 4.2.1 Metacognitive awareness

This study employed a combination of subjective and objective measures to assess students' metacognitive levels. For subjective assessment, we adopted the metacognitive awareness inventory (MAI) as the foundational instrument, contextualizing specific items to the programming domain to ensure content validity (Schraw and Dennison, 1994). For instance, the general construct of “making a plan” was operationalized as “writing pseudocode or decomposing subtasks,” while “self-correction” was defined as “code debugging based on error tracebacks.” The adapted scale comprises three core dimensions: planning, monitoring, and regulation. Each dimension consists of six items (18 items total), as detailed in Table 2. Responses were recorded on a 5-point Likert scale (1 = “completely disagree,” 5 = “completely agree”). To facilitate statistical analysis and comparability, raw scores (maximum 90) were normalized to a 0–100 scale.

**Table 2:** Modified MAI for programming.

Dimension	No.	Item content
Planning	1	I carefully read the problem statement to identify specific input and output requirements before writing any code.
	2	I break down complex programming problems into smaller tasks or modules.
	3	I organize my logic using pseudocode, flowcharts, or scratch paper before typing.
	4	I think about similar problems I have solved before to see if I can apply existing algorithms or patterns.
	5	I estimate the time required for different functional modules and schedule my programming progress accordingly.
	6	I set clear goals for the programming task.
Monitoring	7	I frequently run completed segments to check if intermediate results are correct.
	8	I pause to ask myself if I am deviating from my original plan when the code logic becomes complex.
	9	I track whether data matches expectations by printing variable values.
	10	I am aware of whether I truly understand specific syntax or library functions, rather than mechanically copying and pasting.
	11	I periodically check my current progress to decide if I need to speed up to finish the task on time.
	12	I can immediately realize that there might be a potential bug.
Regulation	13	When encountering an error, I read the traceback carefully to locate the cause before modifying the code.
	14	I will decisively abandon the current approach and try other algorithms or structures if it does not work.
	15	When stuck, I can accurately extract keywords to search for answers in search engines or documentation.
	16	After solving a problem, I reflect on whether the error was caused by a logic flaw or carelessness.
	17	Once the code runs successfully, I try to refactor it to make it more concise or efficient.
	18	I check if the final results cover all boundary conditions (e.g., empty inputs, extreme values).

Given that the original MAI items were contextualized for a programming environment, an exploratory factor analysis (EFA) using principal component analysis with Varimax rotation was conducted to evaluate construct validity. The Kaiser–Meyer–Olkin (KMO) measure verified the sampling adequacy for the analysis (*KMO* = 0.83, *p* < 0.001), and Bartlett's test of sphericity was highly significant (χ^2^ = 1087.42, *p* < 0.001). The analysis extracted three distinct factors: planning, monitoring, and regulation, which cumulatively explained 61.3% of the total variance. All 18 items exhibited strong primary factor loadings ranging from 0.50 to 0.81. While four items exhibited moderate cross-loadings between the monitoring and regulation dimensions, this overlap is theoretically sound within the SRL framework, as monitoring an error state frequently triggers regulatory actions, which in turn iteratively feed back into subsequent monitoring. Coupled with the robust internal consistency (Cronbach's α = 0.88), these results confirm that the modified instrument is highly valid and reliable for this study. The detailed exploratory factor analysis results are provided in Appendix A.

#### 4.2.2 Log-based behavioral metrics

To mitigate the inherent subjective and bias of self-reported inventories, we extracted key indicators reflecting metacognitive behaviors from their objective programming logs. This operationalization of abstract metacognitive phases into log-based indicators is grounded in the trace-based measurement paradigm of SRL (Winne and Nesbit, 2010). This paradigm posits that learners leave objective “traces” when engaging in covert cognitive tactics, which can be computationally operationalized. By mapping the three core components of metacognitive strategies (planning, monitoring, and regulation) onto interaction logs from IDEs or online platforms, this approach allows us to objectively characterize and quantify students' metacognitive behavioral patterns. As outlined in Table 3, we operationalized abstract metacognitive behaviors into quantifiable metric:

- Planning: primarily manifested in the pre-coding phase, this involves thinking and architectural design efforts. Previous studies have shown that high-performing learners tend to spend a longer period on problem understanding and planning to construct a mental mode, whereas novices often skip this phase (Lister et al., 2004; Liew, 2005). Therefore, metrics such as “first edit latency,” “reading dwell time,” and “comment-code ratio” serves as measurable proxies for strategic planning.
- Monitoring: this entails the real-time supervision and assessment of ongoing cognitive processes. Metrics include “execution frequency,” “error dwell time,” and “consecutive error rate.” This study algorithmically distinguishes between effective and ineffective monitoring. A balanced interval indicates effective progress checking. Conversely, extremely short intervals are classified as ineffective monitoring, suggesting “blind trial-and-error” that leads students down and unproductive paths (Jadud, 2006).
- Regulation: occurring after monitoring detects deviations, regulation manifests as a correction of cognitive strategies. This phase is quantified through metrics such as “debugging location accuracy” to evaluate the spatial precision of corrective actions, and “ineffective trail rate” to identify maladaptive “blind” retries during the debugging process. These indicators adapt established learning analytics metrics that evaluate the efficacy of code state transitions in response to bugs (Keuning et al., 2018).

**Table 3:** Operationalization of metacognitive behaviors in programming logs.

Dimension	Sub-dimension	Behavioral indicator	Operationalized metric
Planning	Orientation	Analyzing requirements and reviewing task documentation	• First edit latency
			• Reading dwell time
	Strategic planning	Drafting pseudocode and defining variables before implementation	• Comment-code ratio
Monitoring	Progress monitoring	Executing code to verify logic against expectations	• Execution frequency
	Error detection	Pausing to analyze error messages and console output	• Error dwell time
			• Consecutive error rate
Regulation	Debugging and fix	Modifying code iteratively to resolve errors	• Debugging location accuracy
			• Total debugging counts
			• Debug success rate
	Strategy shifting	Refactoring code or restructuring logic after reflection	• Code deletion volume
			• Ineffective trial rate

Each log record comprises essential fields including timestamp, event type, and detailed parameters. These logs were formatted as structured text (e.g., CSV or JSON) to facilitate systematic analysis. Table 4 presents an example of the core components of a single log entry. Drawing upon this example, the log documents an instance where Subject S_051 encountered an IndentationError in Task_03_Recursion. Through this robust design, we can trace the programming process, establishing a solid basis for comparing metacognitive behavioral differences between the two groups.

**Table 4:** Schema and description of programming process logs.

Field name	Description	Example	Derived metrics
User_ID	Anonymized unique identifier for the student	S_051	Grouping variable
Problem_ID	Identifier for the specific programming task	Task_03_Recursion	Task boundary
Timestamp	Exact date and time of the event	2025-05-20 14:30:45	Temporal metrics
Event_Type	Category of interaction (e.g., run, save, edit)	Run_cell	Behavioral frequency
Code_Source	Snapshot of the code content at the time of event	21: for *i* in range (10):	Effort metrics
		22: print(*i*)	
		23: print(“Done”)	
		24: *x* = *x* + 1	
Exec_Status	Outcome of the code execution	Error	Performance metrics
Error_Type	Specific class of exception raised	IndentationError	Error distribution
Error_Details	Structured error message and traceback info	SyntaxError: unexpected EOF	Reflection depth
Cell_Index	Index of the active notebook cell	2	Attention shift

#### 4.2.3 Academic performance evaluation

To ensure the objective evaluation of programming outcomes, submitted codes were graded on a 100-point scale using a standardized rubric. Based on the task requirements and established programming assessment standards in the syllabus, the rubric evaluated four specific dimensions: functional accuracy (60%), code structure and readability (20%), robustness (10%), and documentation and comments (10%). The detailed criteria for each dimension are presented in Table 5.

**Table 5:** Multi-dimensional rubric for the programming assessment (100-point scale).

Dimension	Description	Weight
Functional accuracy	Correct implementation and test case passing	60%
Code structure and readability	Modularity, naming conventions, clarity	20%
Robustness	Boundary cases and exception handling	10%
Documentation and comments	Logical annotation and explanation	10%

Furthermore, to eliminate subjective bias, all submissions were anonymized prior to grading. Two experienced instructors, who were blind to the students' group assignments, independently graded a random subset (30%) of the submissions. The inter-rater reliability was calculated using the intraclass correlation coefficient (ICC, two-way mixed, absolute agreement). The resulting *ICC* = 0.81 indicates good reliability. Discrepancies in the subset were resolved through discussion, and the main rater proceeded to grade the remaining assignments using the aligned standard.

### 4.3 Data analysis

**Figure 3:**
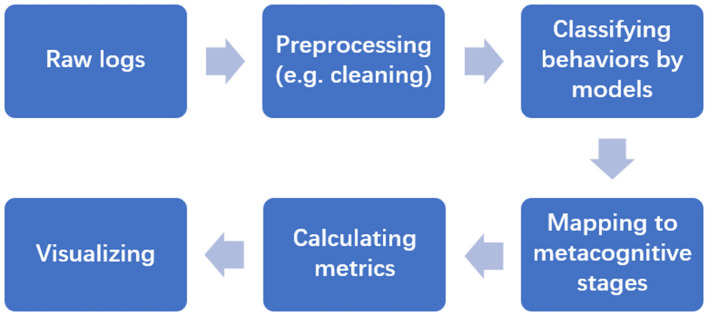
Flowchart of the analysis process from logs to behavior. This flowchart illustrates the data processing stages, demonstrating how unstructured raw log data is preprocessed, classified, and operationalized into quantifiable metacognitive indicators for statistical analysis.

As illustrated in Figure 3, the unstructured log data underwent a rigorous processing pipeline to ensure data integrity and validity:

Prior to feature extraction, a systematic preprocessing phase was conducted to eliminate noise and ensure data quality. First, the raw dataset was filtered to exclude logs generated by teaching assistants, test accounts, and students who withdrew from the course. Second, we applied a “session vitality check” to remove invalid learning sessions; specifically, distinct interaction periods lasting less than 1 min or containing fewer than five active events were classified as “noise” and discarded, as they fail to reflect genuine cognitive engagement. Finally, timestamp irregularities caused by potential network latency were standardized to strictly monotonic sequences to ensure the temporal precision of subsequent latency metrics.

To operationalize abstract behavioral patterns into quantifiable metrics, we extracted key indicators directly from the unstructured log data. In terms of planning strategies, we calculated First Edit Latency, defined as the temporal interval between task initiation (*T*_*start*_) and the first code entry (*T*_*first*_*edit*_), alongside comment-code ratio, which utilizes regular expressions to measure the proportion of annotations relative to total code volume. Regarding monitoring, we computed error dwell time—the average duration elapsed between an error report and the subsequent edit—to capture the depth of error analysis. Furthermore, the regulation metrics required strict thresholding: debugging location accuracy was determined by verifying whether subsequent code edits fell within a ±3 line threshold of the reported error source. Finally, ineffective trial rate was computed by capturing consecutive execution errors where the Levenshtein edit distance between the mode modifications was strictly less than 3, mathematically quantifying blind retries.

To computationally extract the regulation metrics from the processed logs, we applied specific algorithmic rules. Debugging location accuracy was determined by verifying whether subsequent code edits fell within a ± 3 line threshold of the reported error source. Furthermore, the ineffective trial rate was computed by capturing consecutive execution errors where the Levenshtein edit distance between the consecutive code modifications was strictly less than 3, which mathematically quantifies “blind” trial-and-error behaviors.

We employed quantitative methods to analyze the data, utilizing Python libraries (specifically Pandas and SciPy) for all processing and statistical computations. Prior to inferential testing, the assumptions for parametric tests were verifies. The normality of continuous dependent variables (i.e., MAI scores, programming scores, and the eleven log-derived behavioral metrics) was assessed using Q–Q plots and the Shapiro–Wilk test. Results indicated that while most metrics approximated a normal distribution, total debugging counts and error dwell time exhibited non-normal right skewness, which is characteristic of count and latency date. Therefore, for these two specific metrics, the non-parametric Mann–Whitney *U* test was employed to accurately compare the group distribution, whereas independent sample *t*-tests were employed for the remaining normally distributed metrics. Homogeneity of variances for the parametric tests was confirmed using Levene's test. Effect sizes were calculated using Cohen's *d*. To rigorously evaluate the study's hypotheses within our specific context, the following statistical procedures were executed:

1. Analysis of subjective awareness: changes in metacognitive awareness were analyzed using a linear mixed model (LMM) with:
  – Time (pre-test vs. post-test) as a within-subject factor;
  – Group as a between-subject factor;
  – Pre-test MAI score included as a covariate.
2. The model estimated fixed effects for group, time, and their interaction. Standardized coefficients (β), standard errors, and *p*-values were reported (α = 0.05).
3. Analysis of metacognitive behaviors (H1): group comparisons across the operationalized log metrics were conducted using either independent *t*-tests or Mann–Whitney *U* tests, depending on their distribution. To strictly control the family-wise error rate associated with multiple comparisons across the eleven behavioral indicators, a Bonferroni correction was applied. The adjusted significance threshold was established at α′ = 0.05÷11 ≈ 0.0045. Therefore, specifically in comparative analysis of log data, only behavioral differences with *p* < 0.0045 were considered statistically significant.
4. Analysis of learning outcomes (H2): to evaluate the impact of AI-assisted scaffolding on programming performance, post-test programming scores were compared between groups using independent *t*-tests (α = 0.05). Furthermore, to quantify the transfer effect from behavioral changes to academic outcomes, a Pearson correlation analysis was conducted to examine the relationship between students' behavior and their programming performance.

## 5 Results

### 5.1 Analysis of baseline equivalence

Of the initial 126 recruited participants, four students dropped out due to personal reasons (attrition rate: 3.2%), yielding a final valid cohort of 122 participants distributed between the experimental (*n* = 62) and control (*n* = 60) conditions. Before evaluating the intervention effects, we strictly assessed baseline equivalence. As detailed in Table 6, independent samples *t*-tests confirmed parity across initial programming fundamentals (*p* = 0.386) and baseline error frequency (*p* = 0.185). Furthermore, to address potential concerns regarding broad academic disparities, we compared the students' prior semester grades in an “introduction to computer science” course as a metric of general academic achievement. The analysis revealed no significant difference between the AI-assisted group (*M* = 80.68, *SD* = 9.98) and the control group (*M* = 80.53, *SD* = 11.01;*p* = 0.791). Regarding metacognitive capacity, we utilized the reconstructed programming-specific MAI, which yields higher predictive validity for specific task performance than general traits. The pre-test MAI confirmed strong homogeneity between the groups (*p* = 0.231). Collectively, these baseline assessments establish a robust foundation for isolating the intervention effects.

**Table 6:** Comparison of baseline characteristics between groups.

Metric	AI-assisted group (n = 62)	Control group (n = 60)	*p*-value
MAI (pre-test)	50.7 ± 6.2	51.9 ± 4.3	0.231
Programming fundamentals score	52.7 ± 9.7	51.0 ± 13.9	0.386
Baseline error frequency	18 ± 6.4	19 ± 7.6	0.185
Prior “introduction to computer science” Grade	80.7 ± 9.9	80.5 ± 11.0	0.791

### 5.2 Academic performance

Following the intervention phase, both groups completed a post-test programming assessment equivalent in difficulty to the intervention tasks, scored on a 100-point scale based on the multi-dimensional rubric established in Section 4.2.3. Post-intervention assessments revealed a distinct performance gap. As detailed in Table 7, the AI-assisted group (*M* = 85.2, *SD* = 9.4) outperformed the control group significantly (*M* = 76.5, *SD* = 10.8), a difference confirmed by *t*-test results [*t*_(120)_ = 4.75, *p* = 0.002] and supported by a robust effect size (*d* = 0.76). This initial result suggests that the AI scaffolding positively impacted students' final programming outcomes, setting the stage for a deeper analysis of the underlying behavioral mechanisms.

**Table 7:** Comparison of pre-test and post-test programming scores between groups.

Phase	Group	*N*	*M*	SD	*p*-value	Cohen's *d*
Pre-test	AI-assisted group	62	68.4	12.1	0.818	0.04
	Control group	60	67.9	11.8		
Post-test	AI-assisted group	62	85.2	9.4	<0.001	0.76
	Control group	60	76.5	10.8		

### 5.3 Evaluation of metacognitive awareness

**Figure 4:**
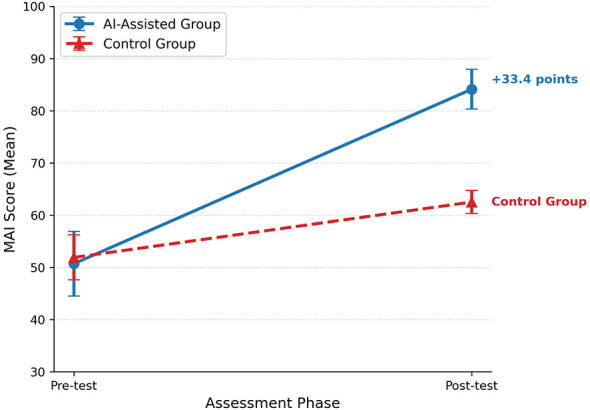
Pre-test and post-test changes in MAI scores. The interaction plot reveals a significant divergence between groups. While baseline scores were comparable, the AI-assisted group (solid blue line) demonstrated a substantial increase (+33.4 points) in self-reported metacognitive awareness compared to the control group (dashed red line), indicating the intervention's efficacy in enhancing subjective metacognitive perception.

To assess the subjective impact of the intervention, we analyzed changes in MAI scores. A linear mixed model, employed to rigorously account for baseline variations, revealed significant differences between the groups (Table 8) (Vickers and Altman, 2001; Bates et al., 2015). The AI-assisted group showed a marked improvement, achieving a mean score of 84.1, which was significantly higher than the control group (*M* = 62.5, Δ_*diff*_ ≈ 21.6). Furthermore, the AI-assisted group demonstrated a substantial pre-to-post gain of 33.4 points, compared to a gain of only 10.9 points in the control group. This confirms that the AI intervention effectively boosted students' self-perceived planning and regulation activities. Interestingly, the pre-test score was not a significant predictor (β = +0.044, *p* > 0.05), suggesting that the final gap was caused by the intervention itself, rather than students' prior abilities. Figure 4 visualizes this shift: despite similar starting points, the AI-assisted group (solid line) shows a sharp increase, providing clear visual evidence that supports our statistical model.

**Table 8:** Results of linear mixed model analysis on post-test MAI scores.

Predictor/group	Post-test mean (*M*)/coefficient (β)	Mean gain (Δ)	Std. error	*p*-value
AI-assisted group	84.1	+33.4	3.8	<0.001
Control group	62.5	+10.9	2.2	<0.001
Pre-test MAI (covariate)	+0.044 (β)	-	0.012	0.174

### 5.4 Analysis of metacognitive behavioral patterns

The comparative results of the objective log metrics are presented in Table 9. Notably, even after applying the strict Bonferroni-corrected threshold (*p* < 0.0045), six out of the 11 behavioral indicators maintained high statistical significance. This robustness confirms that the observed differences were not due to Type I errors derived from multiple comparisons.

**Table 9:** Comparison of metacognitive behavioral metrics between groups.

Metacognitive Indicators	AI-assisted group (n = 62)	Control group (n = 60)	Test statistic (*t*/*U*)	Effect size
	*M (SD)*			***d*** **/** ***r***
First edit latency (s)	186.4 (45.2)	45.3 (18.6)	22.45^***^	4.08
Reading dwell time (s)	125.4 (35.2)	118.6 (40.1)	0.99 (*p* = 0.324)	0.18
Comment-code ratio (%)	15.2 (4.1)	3.5 (1.2)	18.32^***^	3.87
Execution frequency (number of times)	22.3 (7.1)	24.1 (8.5)	−1.27 (*p* = 0.206)	−0.23
Error dwell time (s)	18.5 (5.4)	6.2 (2.1)	3578^U***^	0.85^r^
Consecutive error rate (%)	18.9 (6.2)	21.4 (7.5)	−2.23 (*p* = 0.022)	−0.42
Debugging location accuracy (%)	72.4 (6.3)	61.6 (7.7)	5.51^***^	1.53
Total debugging counts (number of times)	14.2 (5.8)	15.6 (6.5)	1563^U^ (*p* = 0.281)	−0.11^r^
Debugging success rate (%)	78.4 (12.5)	55.2 (15.8)	9.02^***^	1.63
Code deletion volume (lines)	45.6 (18.2)	48.9 (22.4)	−0.89 (*p* = 0.375)	−0.16
Ineffective trial rate (%)	12.4 (5.6)	35.8 (10.2)	−15.84^***^	−2.87

Statistical significance for these eleven behavioral comparisons was determined using a strict Bonferroni-corrected threshold of α′ = 0.0045. For variables satisfying normality assumptions, independent samples *t*-tests and Cohen's *d* were reported.

^U,r^Due to right-skewed non-normal distributions, the Mann–Whitney *U* test was applied, reporting the *U* statistic and non-parametric effect size r.

*** *p* < 0.001.

### 5.5 Planning phase

In the planning phase, we analyzed first edit latency to assess strategic preparation. Results indicated a significant delay in the AI-assisted group's start time compared to the control group. While control participants typically commenced coding immediately (*M* = 45.3 *s*), the AI-assisted group spent significantly longer preparation time [*M* = 186.4 *s, t*_(120)_ = 22.45, *p* < 0.001] before their first edit. This statistically significant delay suggests that the AI agent successfully inhibited the “rush-to-code” impulse, encouraging a more deliberate planning process.

#### 5.5.1 Monitoring and regulation phase

The two groups exhibited divergent behavioral patterns during the monitoring and regulation phases, which are the stages where AI-assisted metacognitive scaffolding intervenes most intensely. This divergence is quantified through key quality-oriented metrics: comment-code ratio, debugging location accuracy, error dwell time, and ineffective trial rate.

Data analysis revealed that the AI-assisted group's mean comment-code ratio was significantly higher than that of the control group [*M*_exp_ = 15.2*% vs*. *M*_*ctrl*_ = 3.5%, *t*_(120)_ = 18.32, *p* < 0.001]. A granular analysis of log content indicates that while control group students typically generated functional code with minimal annotation, AI-assisted group students demonstrated a propensity for drafting logic via comments prior to implementation—influenced by scaffolding prompts.

Regarding debugging location accuracy, the AI-assisted group achieved a superior accuracy rate of 72.4% in locating error sources, compared to 61.6% in the control group [*t*_(120)_ = 5.51, *p* < 0.001]. This advantage was particularly pronounced for logical errors. Logs from the control group showed that approximately 25% of modifications occurred far from the actual error locus, suggesting a failure to correctly interpret error messages. Conversely, the AI-assisted group, aided by the structured error visualization on the AI dashboard, effectively pinpointed problematic areas. Furthermore, the error dwell time differed significantly. When encountering consecutive runtime errors, control students averaged a mere 6.2 s before modifying code, implying a lack of deep analysis. In contrast, the AI-assisted group averaged 18.5 s. This significant difference (Mann–Whitney *U* = 3578, *p* < 0.001) aligns with our design expectation: the AI agent's prompt “consecutive errors detected, recommend checking logic” successfully induced a thinking strategy, slowing down the diagnosis process for better precision.

Correspondingly, the ineffective trial rate for the AI-assisted group (*M* = 12.4%) was significantly lower than that of the control group [*M* = 35.8%, *t*_(120)_ = −15.84, *p* < 0.001]. This indicates that while the control group relied heavily on “blind” trial-and-error, the AI-assisted group exhibited more precise and deliberate debugging behaviors. Notably, the exceptionally large effect sizes observed in metrics such as the comment-code ratio (*d* = 3.87) and ineffective trial rate (*d* = −2.87) are a direct consequence of the system's structured scaffolding design. Rather than representing purely natural behavioral variance, these magnitudes reflect students' high responsiveness to the real-time prompts, which physically interrupted maladaptive habits (e.g., pausing rapid retries) and consistently elicited the targeted metacognitive behaviors.

**Figure 5:**
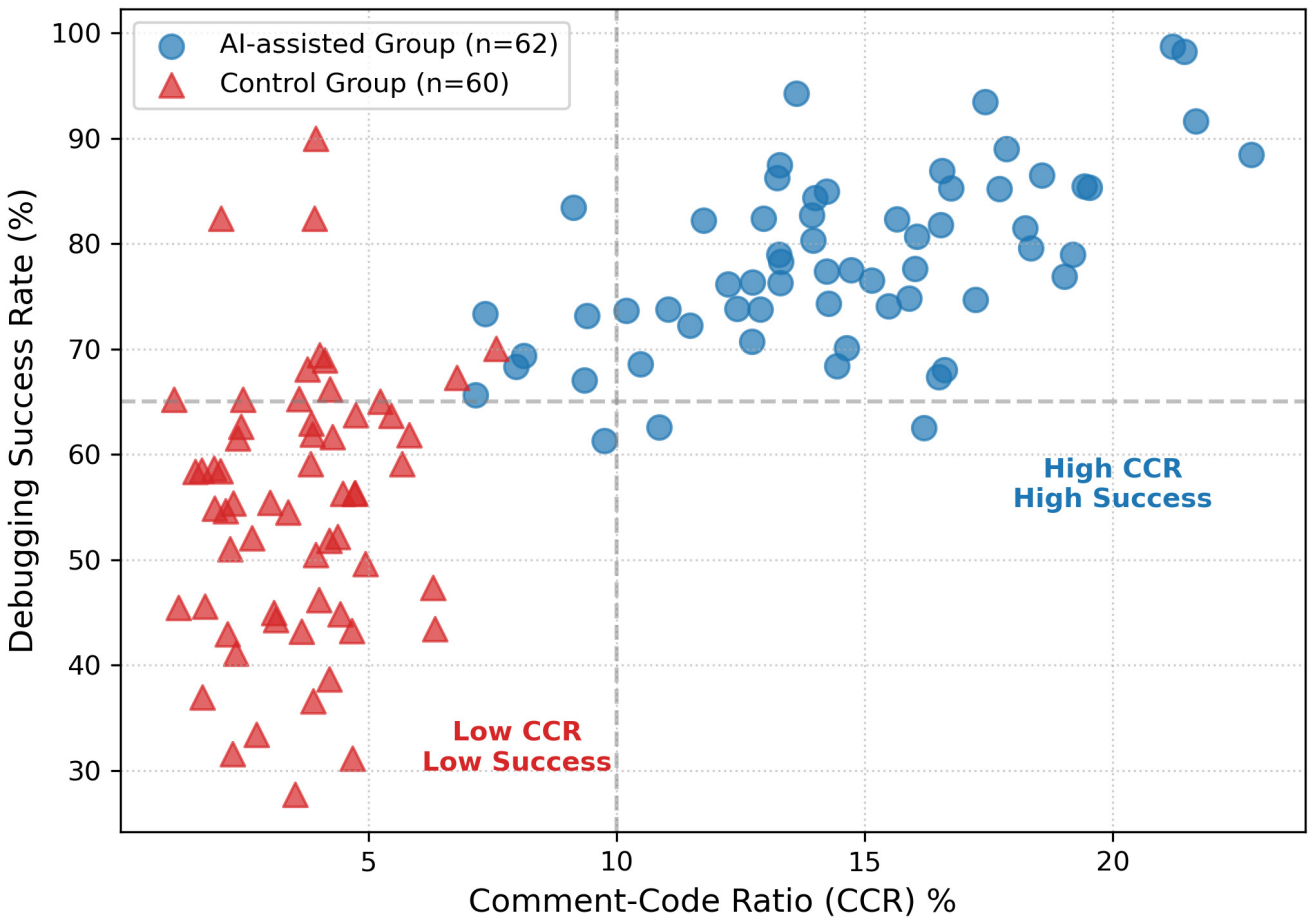
Scatter plot correlating comment-code ratio with debugging success rate (Pearson's *r* = 0.768, *p* < 0.001). The visualization reveals two distinct behavioral clusters. The AI-assisted group (blue circles) clusters in the upper-right quadrant, indicating a positive association between proactive planning (high annotation volume) and debugging success. Conversely, the control group (red triangles) clusters in the lower-left, associating low planning with ineffective trial-and-error.

Figure 5 presents a scatter plot illustrating the relationship between annotation habits (*x*-axis: comment-code ratio) and debugging efficiency (*y*-axis: debugging success rate). Statistical analysis confirmed a significant positive correlation between these two variables across all participants (*r* = 0.768, *p* < 0.001). The data points reveal distinct clusters: the AI-assisted group (blue dots) is predominantly concentrated in the upper-right zone, demonstrating a strong positive correlation between high comment-code ratio and high debugging success rate. Conversely, the control group (red triangles) clusters in the lower-left zone, indicating that a lack of planning is frequently associated with compromised debugging efficiency. Collectively, these data confirm that the AI-assisted group demonstrated superior planning, monitoring, and regulation capabilities. These findings are strongly support H1.

Interestingly, while the AI-assisted group significantly outperformed the control group in both success rate and positioning accuracy, their total debugging counts remained statistically similar (Mann–Whitney *U* = 1563, *p* = 0.28). To provide a comprehensive overview, we analyzed all 11 operationalized indicators, and the results showed that other basic volume and frequency metrics also exhibited no significant differences between the two groups. Under the strictly updated Bonferroni-corrected threshold (*p* < 0.0045), metrics reflecting pure behavioral quantity—such as reading dwell time (*p* = 0.324), execution frequency (*p* = 0.206), and code deletion volume (*p* = 0.375)—showed no statistically significant differences. Even the consecutive error rate (*p* = 0.022) did not meet the adjusted significance threshold. These findings are critical as they reveal that the AI intervention did not simply increase or decrease the sheer number of operations. For example, both groups recorded similar execution frequencies. However, when interpreted alongside the ineffective trial rate, it indicates that the control group frequently executed code for rapid trial-and-error, while the AI-assisted group executed code for deliberate testing. Therefore, the AI-assisted group performed better not because they made more attempts, but because their attempts were more accurate and effective. This confirms a fundamental shift in behavioral strategy: the scaffolding transformed the learning process from quantity-driven trial-and-error into quality-driven, accurate verification.

**Figure 6:**
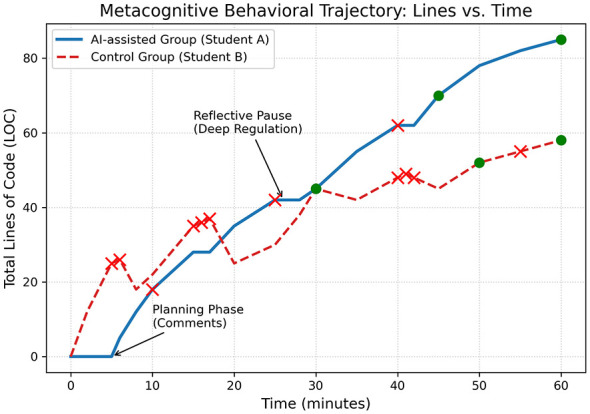
Comparative temporal trajectory of programming behaviors between representative students. The graph contrasts the learning processes of two representative students over a 60-min task. Student A (AI-assisted, solid blue line) exhibits a “step-wise” growth pattern characterized by an initial planning phase and reflective pauses (flat lines) during debugging. In contrast, Student B (Control, dashed red line) shows a volatile “oscillating” pattern with frequent, ineffective code modifications (indicated by red crosses), reflecting a lack of metacognitive monitoring.

Figure 6 illustrates the divergent programming trajectories of two representative learners over a 60-min task. Student A (solid blue line) demonstrates a structured “planning-execution-reflection” pattern. The curve's smooth upward trend and the initial 5-min “zero-code” period reflect a deliberate planning phase. Notably, a 3-min plateau (*Time* = 2528) following an error indicates a “stop-and-think” strategy, where the student paused to analyze traceback information rather than keeping editing—a hallmark of effective metacognitive monitoring.

In sharp contrast, Student B (dashed red line) exhibits high instability. The immediate linear rise in code volume suggests a lack of planning. Significant fluctuations have repeatedly appeared (e.g., *Time* = 58, 2025), signifying large-scale deletion and rewriting. Furthermore, dense clusters of red crosses at *T* = 1517 and *T* = 4042 expose an inefficient trial loop, where the student attempted rapid, luck-based fixes without effective error attribution.

The structural divergence between the two trajectories confirms our hypothesis: the AI-assisted group's higher code volume stemmed from comprehensive documentation and logical structure, rather than redundant code bloat. In contrast, the control group's volatile fluctuations signaled a lack of effective regulation. Ultimately, this proves that AI scaffolding promotes active metacognitive engagement, enabling learners to proactively manage their planning, monitoring, and regulation cycles.

### 5.6 Correlation between metacognitive behavior and academic performance

Although the previous sections established that the AI-assisted group achieved both superior metacognitive behavioral patterns and higher post-test scores, a direct correlation analysis is required to verify the near-transfer effect: whether the improved behaviors actually translated into enhanced programming abilities. To operationalize this, we selected two representative, result-oriented metrics that encapsulate the core phases of the SRL cycle: comment-code ratio (representing the planning phase) and debugging success rate (representing the combined efficacy of the monitoring and regulation phase).

As detailed in Table 10, Pearson correlation analyses across the entire cohort (*N* = 122) revealed significant positive correlation. Both the comment-code ratio (*r* = 0.51, *p* < 0.001) and the debugging success rate (*r* = 0.41, *p* < 0.001) were strongly associated with post-test programming scores. To rule out potential group artifacts and further unpack the intervention's mechanism, subgroup analyses were also conducted.

**Table 10:** Pearson correlations between metacognitive behaviors and post-test performance.

Indicators	Total cohort (*N* = 122)	AI-assisted group (*n* = 62)	Control group (*n* = 60)
Comment-code ratio (planning)	0.51^***^	0.47^***^	0.41^**^
Debugging success rate (monitoring and regulation)	0.41^***^	0.28^*^	0.19^n.s.^

Pearson correlations are reported.

* *p* < 0.05,

** *p* < 0.01,

*** *p* < 0.001, n.s., not significant.

The data revealed a theoretically profound divergence. While planning behavior (comment-code ratio) consistently correlated with higher scores in both groups, the impact of debugging behaviors differed. In the AI-assisted group, debugging success rate was significantly correlated with post-test achievements (*r* = 0.28, *p* < 0.05), indicating a small-to-moderate association. Conversely, in the control group, this correlation was weak and statistically non-significant (*r* = 0.19, *p* = 0.141).

This correlational contrast highlights a fundamental difference in how the two groups engaged with the tasks. In the AI-assisted group, successful debugging was highly predictive of overall programming competence (as measured by the isomorphic post-test), whereas in the control group, resolving immediate errors did not reliably translate to higher academic performance. These correlational findings provide direct statistical evidence for H2, confirming that the intervention successfully facilitated a near-transfer effect.

## 6 Discussion

The results of this study substantiate that AI-assisted metacognitive scaffolding significantly enhances students' metacognitive behaviors in the short term, thereby validating the integration of such scaffolding into programming education and supporting hypotheses H1 and H2. Through a granular analysis of programming logs and performance metrics, we observed a marked increase in the frequency of metacognitive activities, alongside a discernible improvement in learning outcomes.

This finding carries significant theoretical weight within the broader field of educational technology. Consistent with previous studies on SRL in computer-based environments, our data confirms that strategic interventions can activate dormant metacognitive awareness (Azevedo and Gašević, 2019; Yu et al., 2025). However, unlike previous studies that predominantly relied on static academic scores or self-reported scales to evaluate GenAI tools, this research provides process-oriented evidence of behavioral transformation (Zhai et al., 2021; Kasneci et al., 2023). The observed shifts—such as proactive planning prior to coding, frequent deliberate monitoring, and timely regulation—indicate that our AI scaffolding successfully internalized these critical habits. The subgroup analysis further revealed that without AI scaffolding, students' debugging often devolves into shallow “trial-and-error” guessing. This aligns with the well-documented phenomenon of “thrashing” in less experienced learners programming (Jadud, 2006). Our study extends this prior work by empirically demonstrating that GenAI, when deployed as an in-the-moment scaffold, can effectively interrupt these maladaptive loops and transform bug-fixing into a meaningful learning opportunity, significantly enhancing near-transfer to novel tasks.

Furthermore, this mechanistic insight offers a constructive perspective on the ongoing debate regarding potential student over-reliance on GenAI. Recent literature has expressed widespread concern that LLMs might act as a cognitive crutches, inadvertently diminishing student agency and problem-solving resilience (Menon, 2025; Tsakeni et al., 2025). This experiment directly challenges this pessimistic view under specific pedagogical conditions. It demonstrates that when AI functions strictly as a metacognitive scaffold—providing strategic prompts rather than direct solutions—it does not diminish student agency. Conversely, the AI acts as a catalyst, reducing frustration while maintaining the essential “cognitive struggle.” The spontaneous planning behaviors observed during the unprompted phases further suggest that external scaffolding strategies are beginning to transform into internal psychological functions.

It is noteworthy that while metacognitive behaviors showed drastic improvement, the increase in programming scores was relatively moderate compared to the behavioral shift. We attribute this discrepancy to a temporal lag effect in skill acquisition. Academic performance is multifactorial; short-term behavioral optimization acts as a precursor to deep learning but may not immediately translate into a quantum leap in test scores. This phenomenon aligns with cognitive load theory (Sweller, 1988; Kirschner, 2002). Adopting new and conscious metacognitive strategies imposes a temporary “germane load.” As noted by researchers investigating the “cost of metacognitive,” this cognitive effort facilitates long-term schema construction but momentarily competes with the working memory required for immediate task execution (Efklides, 2011; Manasia, 2015). We hypothesize that the scaffold first optimizes behavioral patterns, which then gradually accumulate into improved competence. This transformation from “behavioral frequency” to “cognitive proficiency” points to a vital direction for future research: unraveling the specific temporal mechanism of how AI-induced behavioral changes translate into competency gains over time.

## 7 Conclusion and limitations

This study innovatively integrates metacognitive theory with Generative AI to construct a scaffolding model for programming education, verifying its efficacy through empirical research. Our contributions are threefold: first, we confirmed the feasibility of decoding implicit metacognitive processes via multimodal log data, shifting the evaluation paradigm from a single “outcome-oriented” metric to a comprehensive “process-oriented” analysis. Second, empirical evidence demonstrates that the autonomous AI-assisted intervention significantly amplifies planning, monitoring, and regulating behaviors, providing a practical solution to the cognitive overload. Third, the study provides an actionable empirical blueprint for the broader fields of AI-assisted learning. It underscores a critical paradigm shift: rather than using GenAI as a product-oriented code generator that “does the thinking for the student,” ideal educational applications should function as process-oriented scaffolds that teach the student how to think. By prioritizing this “learning how to learn” approach, we demonstrated a scalable path for delivering personalized metacognitive guidance in complex domains like programming.

While promising, interpretation of these results requires caution due to several limitations, which also chart the course for future inquiry. First, the study was confined to a specific cohort and task type with a relatively small sample size, necessitating further validation across diverse demographics (e.g., K-12) and complex learning scenarios to ensure generalizability. Second, whereas log data offers objective insights, it remains an indirect proxy for cognitive processes. Some subtle cognitive activities may have been overlooked, and future research could integrate physiological metrics to triangulate these findings. Furthermore, while we utilized both subjective and objective measures to evaluate the intervention, this study analyzed them as independent evidence streams. Future research could benefit from a more granular correlational analysis to examine the degree of congruence between students' self-perceived metacognitive awareness and their actual enacted behaviors. Finally, while this study successfully verified the immediate correlation between behavioral improvements and academic performance (representing “near transfer”), it did not structurally analyze the long-term “far transfer” effect. Because of the 21-day intervention limit, it remains unknown whether these metacognitive habits will persist after the AI scaffold is entirely faded, or whether they can transfer to entirely different programming language. These remaining questions regarding long-term internalization and cross-domain transfer pathways constitute the primary focus of our subsequent research. Investigating this “calibration accuracy” would offer deeper insights into the mechanism of the awareness-behavior gap.

## Data Availability

The raw data supporting the conclusions of this article will be made available by the authors, without undue reservation.

## Appendix A

**Table A1:** Exploratory factor analysis results of the modified MAI (*N* = 122).

Item	Factor 1: planning	Factor 2: monitoring	Factor 3: regulation
1	**0.81**	–	–
2	**0.74**	–	–
3	**0.71**	–	–
4	**0.50**	–	–
5	**0.63**	–	–
6	**0.76**	–	–
7	–	**0.72**
8	–	**0.59**	0.44
9	–	**0.69**	–
10	–	**0.73**	–
11	–	**0.61**	0.51
12	–	**0.65**	–
13	–	0.45	**0.63**
14	–	–	**0.72**
15	–	0.50	**0.62**
16	–	–	**0.69**
17	–	–	**0.74**
18	–	–	**0.77**

Loadings below 0.44 are suppressed. Bold values in the table indicate the primary factor loadings for the corresponding items.
